# Physiotherapy students’ and officers’ knowledge, attitudes, and perceptions of mental health

**DOI:** 10.4102/hsag.v29i0.2627

**Published:** 2024-09-05

**Authors:** Marilyn Hooblaul, Oladapo M. Olagbegi, Thayananthee Nadasan

**Affiliations:** 1 Discipline of Physiotherapy, College of Health Sciences, University of KwaZulu-Natal, Durban, South Africa

**Keywords:** mental health, physiotherapy students, knowledge, attitude, perceptions

## Abstract

**Background:**

Physiotherapy in mental health is not very popular among physiotherapists, students, healthcare professionals, and people living with mental illness (PLWMI), although many PLWMI are managed by physiotherapy students and physiotherapists.

**Aim:**

This study aimed to determine the knowledge, attitudes, and perceptions of physiotherapy students and physiotherapy community service officers (PCSOs) towards mental health.

**Setting:**

The study was conducted in KwaZulu-Natal (KZN).

**Methods:**

A concurrent mixed-method was employed using the Attitudes Toward Psychiatry (ATP-30) questionnaire and focus group discussions. The questionnaire was distributed to 100 PCSOs and 191 physiotherapy students. Focus groups were conducted to assess the knowledge gained through the undergraduate programme, comprising three groups of students from second to fourth year and two groups of PCSOs. An interview was also conducted with one PCSO.

**Results:**

A total of 146 students and 72 community service officers completed the questionnaire. Participants had an overall positive attitude, with a mean ATP-30 score of 108.02 (standard deviation [s.d.] = 10.86). Those with a family member or friend with a mental illness scored higher. Focus groups revealed limited knowledge about mental health and reluctance to work in the field.

**Conclusion:**

Physiotherapy students and PCSOs in KZN had positive attitudes towards mental health despite limited knowledge. They expressed a desire for more information about mental health.

**Contribution:**

The curriculum needs to include adequate mental health content to prepare students to manage PLWMI after graduation, reducing stigma, and negative perceptions, and ensuring confidence.

## Introduction

According to Global Mental Health Statistics, approximately 970 million people worldwide grapple with mental illness or substance abuse (HopeChest 2019). In Africa, about 29.019 million individuals suffer from depression (Gbadamosi et al. 2022). A recent national survey in South Africa revealed that over 75% of its population likely live with depression (Craig et al. 2022). Given its status as a lower- and middle-income nation marked by substantial income disparity, those experiencing poverty and poor mental health are at an increased risk of remaining impoverished, thereby perpetuating a cycle of intergenerational effects.

Mental health, as defined by the World Health Organization (WHO 2022), denotes the state of an individual capable of effectively coping with life stressors, maintaining a healthy work-life balance, and actively contributing to their community. Conversely, mental illness or disorder, as per WHO (2022), occurs when an individual experiences disturbances in cognition, emotion, and behaviour that are diagnosable conditions. It is important to observe that poor mental health does not necessarily equate to a diagnosable mental illness. Factors such as burnout, stress, mental exhaustion, grief, or physical conditions can contribute to poor mental health. Stigma transcends mere conceptual or attitudinal barriers; it can profoundly impact both the body and mind (Elmer 2023). People living with mental illness (PLWMI) often contend not only with their disorder but also with the associated discrimination and prejudice (Probst & Skjaerven 2017). Physiotherapists must remain cognisant of discrimination and negative perceptions when caring for PLWMI, as these factors can significantly influence treatment outcomes and the quality of care provided. Combatting stigma necessitates a concerted effort to raise awareness (Probst & Skjaerven 2017).

Physiotherapy offers numerous benefits for individuals with serious mental illness, yet research indicates a need for greater integration of physical health programmes into clinical practice (Probst & Skjaerven 2017). The mental health advantages of physiotherapy encompass enhanced mood, sleep patterns, cognitive function, self-esteem, and overall improved quality of life (Kaur & Garnawat 2009).

Physiotherapists play a vital role in mental health as part of a multidisciplinary team (Kaur & Garnawat 2009). Their management complements medication and psychotherapy, spanning from musculoskeletal disorders to pain management, strength and joint range, relaxation techniques, endurance, balance, and gait through individually tailored exercise programmes. Physiotherapeutic interventions encompass exercise, physical activity, relaxation therapy, basic body awareness therapy (BBAT), and touch therapy (Probst & Skjaerven 2017). Among the most prevalent forms of exercise are aerobic and strength exercises, with physiotherapists adept at designing both individual and group exercise programmes for PLWMI (Stathopoulos 2020). Aerobic exercises such as walking, jogging, cycling, and swimming are commonly recommended (Kaur & Garnawat 2009). Relaxation therapy, which reduces stress and anxiety by alleviating tension and fostering a tranquil state of mind, is also pivotal. Techniques such as tai chi, yoga, mindfulness-related exercises, and Pilates aid PLWMI in managing stressful situations (Probst & Skjaerven 2017). Basic body awareness therapy, a technique utilising human movement, focuses on posture, coordination, unrestricted breathing, and awareness, serving as a series of exercises that promote holistic well-being (Probst & Skjaerven 2017). Physiotherapists have identified psychological benefits in patients with eating disorders through physiotherapeutic intervention (Soundy et al. 2016). Educating patients on the bio-psychosocial advantages of physical activity constitutes a key role for physiotherapists (Soundy et al. 2014).

A wealth of research underscores the benefits of physiotherapy in mental health. However, challenges persist regarding the knowledge, attitudes, and perceptions of both physiotherapy students and professionals towards mental health. Eight studies have explored the attitudes and perceptions of physiotherapy students regarding mental health and psychiatry, with only one conducted in South Africa and the remainder internationally (Bhise et al. 2016; Connaughton & Gibson 2016b; Dandridge et al. 2014; Gunduza et al. 2023; Probst & Peuskens 2010; Sarin 2003; Yildirim et al. 2015; Zechner et al. 2022). All eight studies concluded the necessity of including mental health content in undergraduate physiotherapy programmes, encompassing courses, lectures, and clinical practice related to psychiatry (Bhise et al. 2016; Connaughton & Gibson 2016b; Dandridge et al. 2014; Gunduza et al. 2023; Probst & Peuskens 2010; Sarin 2003; Yildirim et al. 2015; Zechner et al. 2022). While physiotherapists may not directly work with PLWMI, they may encounter patients with psychiatric comorbidities alongside other chronic conditions, underscoring the necessity for basic physiotherapeutic treatment skills to manage such cases (Probst & Peuskens 2010; Yildirim et al. 2015).

In South Africa, a study revealed that physiotherapists exhibited limited knowledge about mental health, although this deficiency did not significantly affect their attitudes but rather their perceptions (Hooblaul, Cobbing & Daniels 2020). Providing theoretical and practical exposure to PLWMI for physiotherapy students was deemed essential to reduce the stigma surrounding mental illness (Hooblaul et al. 2020). Moreover, research demonstrates that mental health training positively impacts the attitudes of physiotherapy students, as evidenced by a study conducted at Cardiff University in the United Kingdom (Sarin 2003). Recommendations include implementing courses, lectures, skills training, and clinical practice related to mental health and psychiatric conditions within both undergraduate and postgraduate physiotherapy curricula worldwide (Almirón et al. 2020; Andrew et al. 2019; Bhise et al. 2016; Connaughton & Gibson 2016b, 2016a; Dandridge et al. 2014; Hooblaul et al. 2020; Prasanna, Kumari & Vigneshwar 2021; Probst & Peuskens 2010; Sarin 2003; Yildirim et al. 2015).

Physiotherapists in the KwaZulu-Natal (KZN) public health sector have exhibited positive attitudes towards mental health and psychiatry, but expressed feeling unprepared to manage PLWMI because of limited knowledge acquired during their undergraduate programmes, indicating the necessity for mental health content inclusion (Hooblaul et al. 2020). It is observed that the more knowledge physiotherapy students possess about mental health disorders, the more their attitude towards managing PLWMI improves (Probst & Peuskens 2010). While studies have delved into the knowledge, attitudes, and perceptions of physiotherapy students regarding mental health, there is a gap in research focusing on physiotherapy community service officers (PCSOs) in this regard, particularly in the KZN province of South Africa. Consequently, this study aims to determine the self-reported knowledge, attitudes, and perceptions of physiotherapy students at the University of KwaZulu-Natal (UKZN) and PCSOs in KZN in 2022 towards mental health.

## Research methods and design

A concurrent mixed-methods study design entails the simultaneous collection and analysis of quantitative and qualitative data (Creswell 2008). This study employed a survey administration of a questionnaire for physiotherapy students and PCSOs, alongside focus group discussions with physiotherapy students and PCSOs. The study employed qualitative data obtained through focus groups to assess participants’ knowledge and perceptions. In addition, quantitative data were collected using the Attitudes Towards Psychiatry (ATP-30) tool to evaluate participants’ attitudes. The research explored how attitudes influence knowledge and perceptions and vice versa. The research was conducted at UKZN with physiotherapy students and within various Department of Health institutions in the province of KZN with PCSOs.

The participants in this study comprised 2nd, 3rd, and 4th-year physiotherapy students at the UKZN, along with PCSOs placed in the province of KZN from the eight universities in the country that offer the undergraduate physiotherapy programme. Sample size calculations were conducted using EPI INFO version 7 (StatCalc) (Centers for Disease Control and Prevention 2019). The minimum sample size for students was determined to be 141 (128 + 12.8), and for PCSOs, it was 80, based on a two-sided confidence level (1-alpha) of 95% and 80% power. The study employed total population sampling, wherein a group of individuals shares a common characteristic (Creswell 2008). It is worth considering that physiotherapy community service constitutes a mandatory year of service for every qualified physiotherapist. The KZN Department of Health employs PCSOs for a duration of 12 months.

The ATP-30 was originally developed to assess medical students’ attitudes towards psychiatry in Canada (Burra et al. 1982). Although widely utilised globally, no specific tool exists for measuring the attitudes of physiotherapy students. However, the ATP-30 has been employed in several studies to gauge the attitudes of both physiotherapists and physiotherapy students (Bhise et al. 2016; Connaughton & Gibson 2016a, 2016b; Hooblaul et al. 2020; Probst & Peuskens 2010). Demonstrating reliability and validity (Burra et al. 1982; Probst & Peuskens 2010), the tool was integrated with the questionnaire alongside demographic information (Burra et al. 1982).

The study employed purposive sampling to select participants for the focus groups, ensuring a diverse representation of students and PCSOs (Nagle & Williams 2006). Specifically, one focus group was conducted for each academic level, ranging from 2nd to 4th year, at the UKZN, resulting in a total of three focus groups. Each focus group session entailed two open-ended questions tailored for the physiotherapy students:

1. What knowledge have you acquired about mental health since registering for the undergraduate programme?
2. What would you like to know about mental health before graduating?

Two focus groups and one interview were conducted with PCSOs. Two open-ended questions were used for the interviews with the PCSOs:

1. What knowledge have you acquired about mental health since graduation?
2. What would you have liked to have known about mental health before graduating?

### Data collection

Completed questionnaires were entered into an MS Excel spreadsheet and analysed utilising SPSS software version 28.0. The normality of the ATP-30 scores was assessed using the Shapiro–Wilk test, which indicated a normal distribution of all data. Categorical socio-demographic variables were summarised using frequency tables and percentages. Meanwhile, mean and standard deviation (or median and quartiles for non-parametric data) were employed to summarise ATP-30 scores. The ATP-30 tool consists of a 30-item Likert-type scale, where participants express agreement or disagreement with statements on a 5-point scale (1: strongly agree, 5: strongly disagree). Higher scores denote a more positive attitude towards psychiatry, with the maximum achievable score being 150 and a neutral score set at 90. Statistical significance was established at *p* < 0.05. The relationship between ATP-30 scores and participant characteristics was examined using a binary regression model.

The responses from the interviews and focus groups were transcribed, coded, and subjected to thematic analysis. A qualitative content analysis approach was employed to scrutinise the data, aiming to identify common themes or concepts. Emerging themes were discerned and categorised. Deductive content analysis was utilised to facilitate comparisons with prior studies investigating the attitudes of physiotherapy students towards mental health. Established criteria outlined in a checklist were adhered to ensure the study’s trustworthiness throughout the content analysis phases (Elo et al. 2014). Themes were derived and verified through extensive deliberations among researchers. Methodological rigour was upheld through member checking and the incorporation of detailed, descriptive accounts. To preserve anonymity, illustrative quotes were attributed with pseudonyms (Creswell & Miller 2000).

### Ethical considerations

The study meticulously adhered to all ethical, legal, and regulatory guidelines established by the relevant authorities at the country, provincial, and departmental levels. Ethical clearance was officially obtained from the University of KwaZulu-Natal Humanities and Social Sciences Research Ethics Committee (Reference number: HSSREC/00004701/2022). The KZN Department of Health Gatekeepers approval was granted (KZ_202207_023).

Each participant was provided with comprehensive information regarding the study and explicitly informed of their voluntary participation rights, including the freedom to withdraw at any juncture. Stringent measures were implemented throughout the research process to uphold confidentiality, anonymity, and privacy. The study was conducted in accordance with the ethical principles delineated in the Helsinki Declaration.

## Results

A total of 218 participants, comprising 146 students and 72 community service officers, completed the questionnaire, representing 75% of the entire population. This response rate exceeded the minimum sample size criteria. Participants provided demographic information and responded to questions regarding personal connections to mental illness and the frequency of treating PLWMI. The age range of participants was 18–46 years, with a mean age of 22 years (standard deviation [s.d.]: 10.9). Table 1 indicates a predominance of female participants across both students and PCSOs, with 76.5% (*n* = 166) being female. Among the student cohort, the majority of third-year students reported treating a patient with a comorbid mental illness (69.6%), while this proportion slightly decreased to 55.6% among fourth-year students. All second-year students indicated no prior experience in treating a patient with a comorbid mental illness, whereas all PCSOs reported having such experience. None of the students reported treating PLWMI daily, while a subset of PCSOs (*n* = 10) indicated daily treatment. Regarding treatment frequency, 37% of third-year and 37.8% of fourth-year students reported treating PLWMI at least once a month, whereas 27.8% of PCSOs reported treating PLWMI approximately 1–2 times a week.

**TABLE 1:** The study characteristics according to the different participant profile.

Participant characteristics	2nd year		3rd year		4th year		PCSO	
	*n*	%	*N*	%	*n*	%	*n*	%
**Gender**								
F	42	76.4	38	82.6	32	71.1	54	75.0
M	13	23.6	8	17.4	12	26.7	18	25.0
Prefer not to say	0	0.0	0	0.0	1	2.2	0	0.0
**Family or friend**								
N	43	78.2	27	58.7	27	60.0	20	27.8
Y	12	21.8	19	41.3	18	40.0	52	72.2
**Treated patient with comorbid mental illness**								
N	55	100.0	14	30.4	20	44.4	0	0.0
Y	0	0.0	32	69.6	25	55.6	72	100.0
**Frequency of treating PLWMI**								
Never (ref)	55	100.0	14	30.4	20	44.4	0	0.0
Once a month	0	0.0	17	37.0	17	37.8	18	25.0
Twice a month	0	0.0	9	19.6	5	11.1	18	25.0
1–2 times a week	0	0.0	4	8.7	2	4.4	20	27.8
3–4 times a week	0	0.0	2	4.3	1	2.2	6	8.3
Everyday	0	0.0	0	0.0	0	0.0	10	13.9

F, female; M, Male; N, no; Y, yes; PLWMI, people living with mental illness; PCSO, physiotherapy community service officer; ref, reference.

Attitude was assessed using the ATP-30 tool appended to the questionnaire. A positive attitude was delineated when participants scored between 91 and 150 on the ATP-30 scale, while a neutral attitude was represented by a score of 90, and a negative attitude was indicated by a score below 90 (Burra et al. 1982). The mean ATP-30 score indicated a positive attitude of 108.02 (s.d. = 10.86). Table 2 illustrates the correlation between ATP-30 scores and participants’ characteristics. Female participants exhibited a more positive attitude compared to male participants (*p* = 0.340), although this difference was not statistically significant. There existed a statistically significant discrepancy (*p* = 0.047) in ATP-30 scores among 2nd and 4th-year students, PCSOs, and 3rd-year students. Specifically, 2nd-year students garnered the highest ATP-30 score (109.65). Participants who reported having a family member or friend with a mental illness demonstrated a more positive ATP-30 score than those lacking such familial connections. Notably, 43.6% of participants had never encountered PLWMI, whereas only 4.6% treated PLWMI daily. Participants who engaged in daily treatment of PLWMI exhibited a higher ATP-30 score than those with no prior experience in treating PLWMI.

**TABLE 2:** Participants’ characteristics and the association of ATP-30 scores by using ANOVA and independent *t*-test.

Participant characteristics	Total		Score		ANOVA/Independent *t*-test *p*-value	Tukey’s Post hoc comparison
	*n*	%	Mean	s.d.		
**Gender**						
F	166	76.1	108.42	9.94	0.172	-
M	51	23.4	106.82	13.52	-	-
Prefer not to say	1	0.5	103.00	-	-	-
**Year**						
2nd years^(a)^	55	25.2	109.65	10.03	0.047*	[d > b]*
3rd years^(b)^	46	21.1	105.07	9.23	-	[c > b]*
4th years^(c)^	45	20.6	108.96	9.21	-	[a > b]*
PCSO^(d)^	72	33.0	108.08	12.99	-	-
**Family or friend**						
No (Ref)	117	53.7	107.04	10.11	0.477	-
Yes	101	46.3	109.16	11.61	-	-
**Treated with co-morbid mental illness**						
No (Ref)	89	40.8	107.93	9.80	0.076	-
Yes	129	59.2	108.09	11.56	-	-
**How often**						
Never (Ref)	95	43.6	107.96	9.74	0.086	-
Once a month	45	20.6	106.40	12.49	-	-
Twice a month	32	14.7	106.94	7.68	-	-
1–2 times a week	27	12.4	108.07	12.18	-	-
3–4 times a week	9	4.1	110.78	12.68	-	-
Everyday	10	4.6	116.80	14.23	-	-

Note: (a) refers to 2nd years, (b) refers to 3rd years, (c) refers to 4th years and (d) is PCSOs. (a), (c) and (d) have a greater ATP 30 score than (b).

PCSO, physiotherapy community service officers; s.d., standard deviation; ANOVA, Analysis of variance; F, female; M, male.

* , *p* < 0.05.

A significant correlation was observed between having provided treatment to a patient with a mental illness and having a family member or friend afflicted with a mental health condition. Participants who reported having family members or friends with mental illness were 6.67 times more likely to have treated PLWMI (Adjusted Odds Ratio [AOR] = 6.67, 95% Confidence Interval [CI] = 3.51–12.49, *p* < 0.000) (refer to Table 3). Gender did not exert any influence on the frequency of treatment of PLWMI. However, both the academic year of study and engagement in community service significantly influenced the likelihood of students or PCSOs treating PLWMI.

**TABLE 3:** Association of treatment of patients with comorbid mental illness with selected participants’ characteristics by using a binary logistic regression model.

Participant characteristics	Treated pt with comorbid MI				Chi-square *p*-value	Adjusted OR	95% CI	*p*
	No		Yes					
	Count	%	Count	%				
**Family or friend**								
No (Ref)	70	59.8	47	40.2	0.000***	-	-	-
Yes	19	18.8	82	81.2		6.67	3.51–12.49	0.000***
**Years of study**								
2nd	55	100.0	0	0.0	0.023*	-	-	-
3rd	14	30.4	32	69.6		1.67	0.71–3.96	0.062
4th (Ref)	19	42.2	26	57.8		-	-	-
PCSOs	0	0.0	72	100.0		-	-	-
**Frequency of treating PLWMI**								
Never	88	92.6	7	7.4	0.041*	-	-	-
Once a month	0	0.0	46	100.0		-	-	-
Twice a month	0	0.0	32	100.0		-	-	-
1–2 times a week	0	0.0	26	100.0		-	-	-
3–4 times a week	0	0.0	9	100.0		-	-	-
Everyday	0	0.0	10	100.0		-	-	-

OR, odds ratio; CI, confidence interval; PCSO, physiotherapy community service officers; PLWMI, people living with mental illness; MI, mental illness; pt, patient.

* , *p* < 0.05;

*** , *p* #x003C; 0.001.

The findings from the focus group discussions are presented in Table 4 and Table 5. Both the students and PCSOs self-reported limited knowledge regarding mental health during the focus groups and interviews. Participants expressed a strong desire for enhanced understanding of mental health topics. The participants in this study demonstrated a basic understanding of mental health disorders. However, they expressed a desire for more physiotherapy-specific knowledge, recognising that physiotherapists play a crucial role in managing PLWMI. This highlights the importance of incorporating specialised mental health content into the physiotherapy curriculum to meet the educational needs and expectations of future physiotherapy practitioners. For instance, A participant articulated:

‘We did psychology in the first year, but we did not learn any practical skills; if a patient has a certain condition, what I can do for them.’ (Participant 1, Focus group 2, 2nd year student)

**TABLE 4:** Responses to the open-ended questions from students.

What have you learned about mental health in undergraduate training?	What would you have liked to know about mental health before graduation?
Understand the different mental health disorders.	Physiotherapy-specific training about mental health. Counselling of patients. Physiotherapy’s role in mental health How to identify psychological distress in patients How to communicate with patients Role of members of the MDT

MDT, multi-disciplinary team.

**TABLE 5:** Responses to the open-ended questions from the physiotherapy community service officers.

What have you learned about mental health in undergraduate training?	What would you have liked to know about mental health before graduation?
Theory about mental health disorders Communication strategies	The link between the body and the mind Role of physiotherapy in mental health Mental health awareness Psychosomatic pain Practical exposure to patients with a mental illness

This sentiment of inadequate mental health knowledge was echoed across multiple focus groups, with one exception where a participant claimed to have received sufficient training in mental health during their undergraduate programme. Physiotherapy community service officer participants highlighted feeling unprepared to manage PLWMI effectively.

The students expressed a unanimous consensus regarding the need for training in communication skills. This highlights the recognition among students of the pivotal role effective communication plays in their interactions with PLWMI and underscores the importance of incorporating communication training into their education, one said:

‘I would have to explain to the patient the same thing over and over, and it was frustrating.’ (Participant 3, Focus group 4, 3rd year student)

Another participant emphasised:

‘I need to know a lot and everything there is to know about mental health.’ (Participant 2, Focus group 2, 4th year student)

The PCSOs shared the sentiment that they would have benefited from clinical exposure to PLWMI as this would have given them the confidence to manage PLWMI. Furthermore, some participants shared their perceptions regarding mental health and PLWMI, such as, a participant remarked:

‘I function on ignorance is bliss, if I see the patient I will exclude that you have a mental illness and treat the rehab problem.’ (Participant 3, Focus group 1, PCSO)

The students stated that negative perceptions and stigma could play a role in the treatment of PLWMI. Another participant stated:

‘I think lack of knowledge and especially surrounding depression, people will think that they are lazy and they don’t want to do anything.’ (Participant 4, Focus group 2, 4th year student)

## Discussion

The research examines the self-reported knowledge, attitudes, and perceptions of physiotherapy students at UKZN and PCSOs in KZN regarding mental health. Analysis of the ATP-30 tool results indicated an overall positive attitude among the participants. Despite reporting limited knowledge acquired during their undergraduate programme, the participants demonstrated a favourable disposition towards mental health. This finding resonates with a similar study conducted among physiotherapists in KZN (Hooblaul et al. 2020).

Knowledge, defined as the ability to act or abstain based on factual understanding (Bolisani & Bratianu 2018), was assessed through focus groups. Participants, both students and PCSOs, indicated receiving psychology lectures in their first year of undergraduate study, but acknowledged having limited knowledge about mental health, mirroring findings from other studies involving physiotherapists (Almirón et al. 2020; Andrew et al. 2019; Dandridge et al. 2014; Hooblaul et al. 2020). Rutvi, Shah and Parikh (2022) and Gunduza et al. (2023) found that physiotherapy students demonstrated moderate knowledge of mental health, with the education system and university curriculum playing pivotal roles in these outcomes (Rutvi et al. 2022). Notably, the study observed an improvement in physiotherapy students’ knowledge from the second year to the fourth year of their undergraduate programme, attributed to clinical exposure to patients beginning in the third year, a pattern consistent with findings from a study at the University of Witwatersrand (Gunduza et al. 2023). Differences in knowledge and attitudes among student groups were linked to clinical placements and seminars (Gunduza et al. 2023), with many students expressing uncertainty about managing PLWMI, despite encountering patients with co-morbid mental health conditions primarily focused on addressing physical ailments. Physiotherapy community service officers, drawn from various universities across South Africa, similarly reported feeling ill-prepared by limited mental health training for their community service year and managing PLWMI. Only one PCSO participant from the focus groups indicated having received comprehensive mental health training, both theoretically and practically, during their undergraduate programme and felt adequately prepared to manage PLWMI post-graduation. Disparities in mental health training within curricula were evident, highlighting a need for standardisation and enhancement in this area.

Bohner and Dickel (2011) define attitude as the evaluation of an object of thought. In this study, participants exhibited moderately positive attitudes. Notably, second-year students lacking clinical experience demonstrated higher ATP-30 scores than PCSOs, a deviation from findings in prior studies indicating a positive correlation between a higher academic year and more favourable attitudes towards mental health (Connaughton & Gibson 2016b; Gunduza et al. 2023; Rutvi et al. 2022). This discrepancy in scores could stem from two potential factors: either second-year students responded neutrally (scoring 3) on the ATP-30 tool or felt compelled to provide socially desirable responses (Probst & Peuskens 2010). Female participants attained higher ATP-30 scores than their male counterparts, consistent with findings from other studies (Connaughton & Gibson 2016a, 2016b; Probst & Peuskens 2010). The disparity in gender ratio among participants may be attributed to physiotherapy being a profession predominantly pursued by females (Hooblaul et al. 2020). Furthermore, the study revealed that having a family member or friend with a mental illness correlated with improved attitudinal scores compared to participants lacking such connections, a trend also observed in prior research (Connaughton & Gibson 2016a; Hooblaul et al. 2020). Interacting with PLWMI on a daily basis was found to positively influence participants’ attitudes, with scores higher among those regularly engaging with PLWMI compared to those who had no prior experience treating them. This finding aligns with studies demonstrating higher ATP-30 scores among physiotherapists who frequently interact with PLWMI (Connaughton & Gibson 2016a; Hooblaul et al. 2020). Moreover, this study identified parallels between the frequency of treating PLWMI and having a family member or friend with a mental illness (Connaughton & Gibson 2016a; Hooblaul et al. 2020). These findings confirm that interactions with family members or friends with mental illness can influence treatment frequency and that limited knowledge does not significantly impact participants’ attitudes towards mental health.

Physiotherapy students reported minimal knowledge about mental health, lacking specific theoretical training in physiotherapy pertaining to mental health, and no clinical exposure to psychiatry or mental health. Despite this, they indicated treating PLWMI in a general setting. A study conducted in Finland demonstrated significant improvement in students’ attitudes towards mental health following an intensive 67-h course on the subject (Probst & Peuskens 2010). Education plays a pivotal role in shaping attitudes and can mitigate stigmatising behaviours through two potential strategies: theoretical instruction and practical exposure. Both the questionnaire and focus groups revealed that physiotherapy students comprehend the role of physiotherapy in mental health and recognise the significance of physiotherapy interventions for PLWMI. However, there appears to be a reluctance among students to work in psychiatric hospitals, potentially stemming from limited knowledge about mental health, a finding consistent with another study conducted in KZN (Hooblaul et al. 2020).

Perception, as defined by Qiong (2017), involves the brain’s interpretation and organisation of sensory information, influenced by personal experiences, emotions, motivations, and expectations. Analysis of perceptions was conducted through focus groups with participants, revealing their lack of confidence in managing PLWMI because of limited knowledge. While participants recognised their role in mental health, they expressed uncertainty about its implementation, lacking practical training in mental health management. The desire for more mental health education during the undergraduate programme suggests that limited knowledge has indeed shaped participants’ perceptions. Despite acknowledging the value of physiotherapy interventions for individuals with mental illness, participants expressed reluctance to work in psychiatric facilities, consistent with findings from a prior study at the University of Witwatersrand (Gunduza et al. 2023). While students viewed working in mental health as prestigious, they deemed mental health education non-essential unless required for degree completion. Many PCSOs admitted feeling unprepared to manage PLWMI post-graduation, a sentiment exacerbated by stigma, potentially contributing to physiotherapy students’ reluctance to engage with PLWMI (Zechner et al. 2022). Given the high prevalence of mental illness in South Africa, it is imperative for physiotherapy students to acquire adequate skills and cultivate a positive attitude towards mental health. Accordingly, the physiotherapy curriculum should incorporate mental health training comprising relevant course content and opportunities for interaction with PLWMI.

### Strengths and limitations

To the authors’ knowledge, this study represents the first investigation into the knowledge, attitudes, and perceptions of physiotherapy students and PCSOs in KZN and only the second such study conducted in South Africa. While the study achieved a satisfactory response rate during the data collection period, challenges arose in recruiting participants because of conflicts in students’ schedules, suggesting potential for further improvement in recruitment strategies. A notable limitation of the study pertains to the use of the ATP-30 tool, originally designed for assessing attitudes among medical and occupational therapy students. Given the absence of a specific tool tailored for measuring attitudes among physiotherapy students, the applicability of the ATP-30 tool may be questioned. This limitation underscores the need for the development or adaptation of assessment tools tailored to the unique context and requirements of physiotherapy education and practice.

### Recommendations

Further research endeavours should aim to identify effective approaches for reducing stigma and enhancing educational outcomes, including the optimal amount and type of training required to equip students with the skills and confidence to effectively work with PLWMI. In addition, studies should be conducted across other universities in South Africa that offer undergraduate physiotherapy programmes to ensure a comprehensive understanding of the challenges and opportunities in mental health education within this context. The findings from this study shed light on significant gaps in mental health content within the physiotherapy curriculum. Students expressed a clear desire for more comprehensive information about mental health, particularly within the context of the physiotherapy profession. Therefore, the study underscores the urgent need for a thorough review of the current curriculum in South Africa, with a specific focus on enhancing mental health training. Such revisions should aim to incorporate relevant and practical content that adequately prepares physiotherapy students to address the mental health needs of their future patients.

## Conclusion

This study revealed that both physiotherapy students at UKZN and PCSOs in KZN self-reported having limited knowledge about mental health during their training. Despite this, the participants demonstrated an overall positive attitude towards mental health. Interestingly, the students reported managing PLWMI with comorbidities in a general setting, indicating their involvement in mental healthcare despite their perceived lack of knowledge. Comparatively, PCSOs reported greater exposure to PLWMI and more frequent interactions with them than the students. However, both groups expressed a need for enhanced skills to effectively manage PLWMI after graduation. Education was identified as a potential means to foster a positive attitude towards mental health and reduce stigma among physiotherapy students and PCSOs. Given these findings, there is a clear imperative for the review of the physiotherapy undergraduate curriculum in South Africa to incorporate comprehensive mental health content. By equipping students with the necessary skills and knowledge, the curriculum can better prepare future physiotherapists to address the mental health needs of their patients effectively.
